# A Woman’s International Provers’ Association

**Published:** 1894-10

**Authors:** Sophia Penfield

**Affiliations:** Secretary


*Secretary.*

---

### A WOMAN'S INTERNATIONAL PROVERS' ASSOCIATION.

A Woman's International Provers' Association was organized at Chicago during the World's Congress. Officers were elected for the year. President, Dr. Martha Canfield; Vice-Presidents, Drs. M. E. Avery, MacCracken, Jane Culver, Marion McMasters, Sarah Millsop, Millie Chapman; Secretary, Sophia Penfield.

A large number of women physicians were interested in the work of proving. An old and familiar remedy, Conium-mac., was selected for the first year's work. Careful instructions relative to her qualification as a prover, and manner of proving, was sent to each member of the Association. Each prover recorded general condition and symptoms for one month before taking the remedy, which was in the 30x, 3x, 1x, and $\theta$. Press of work and other adverse influences caused an abatement of interest among the provers, but seven full reports were presented at the annual meeting at Denver.

The provings were more interesting, as a promise of future work by women, than for any additional symptoms obtained. The two symptoms, sharply emphasized by the majority of provers, regardless of attenuation, were: "Dull, occipital headache on rising in the morning, and continuous through day," and "dull ache in lumbar and sacral region." "Depression, vertigo on rising, soreness of eyeballs, with orbital pain, colic, with loose stools, numb aching, with trembling of limbs" were also accented.

At the jubilee meeting at Denver a resolution was adopted to elect officers annually, and to affix the annual fee at $1.00. The officers elected were: President, Dr. Millie Chapman; Vice-President, Dr. Julia H. Smith; Secretary and Treasurer, Sophia Penfield. The vice-presidents appointed for each State at the World's Congress remain in office during the year. The aid of every woman interested in the work is solicited to forward it.

SOPHIA PENFIELD, *Secretary.*

